# Application of Flexible Four-In-One Microsensor to Internal Real-Time Monitoring of Proton Exchange Membrane Fuel Cell

**DOI:** 10.3390/s18072269

**Published:** 2018-07-13

**Authors:** Chi-Yuan Lee, Chia-Hung Chen, Chao-Yuan Chiu, Kuan-Lin Yu, Lung-Jieh Yang

**Affiliations:** 1 Department of Mechanical Engineering, Yuan Ze Fuel Cell Center, Yuan Ze University, Taoyuan 320, Taiwan; s1010927@mail.yzu.edu.tw (C.-Y.C.); s1030943@mail.yzu.edu.tw (K.-L.Y.); 2 Homytech Global Co., Ltd., Taoyuan 334, Taiwan; sv3@homytech.com; 3 Mechanical and Electromechanical Engineering, Tamkang University, Taipei 251, Taiwan; ljyang@mail.tku.edu.tw

**Keywords:** PEMFC, MEMS, real-time diagnostic tool, flexible four-in-one microsensor

## Abstract

In recent years, the development of green energy sources, such as fuel cell, biomass energy, solar energy, and tidal energy, has become a popular research subject. This study aims at a flexible four-in-one microsensor, which can be embedded in the proton exchange membrane fuel cell (PEMFC) for real-time microscopic diagnosis so as to assist in developing and improving the technology of the fuel cell. Therefore, this study uses micro-electro-mechanical systems (MEMS) technology to integrate a micro humidity sensor, micro pH sensor, micro temperature sensor, and micro voltage sensor into a flexible four-in-one microsensor. This flexible four-in-one microsensor has four functions and is favorably characterized by small size, good acid resistance and temperature resistance, quick response, and real-time measurement. The goal was to be able to put the four-in-one microsensor in any place for measurement without affecting the performance of the fuel cell.

## 1. Introduction

It has been suggested that the internal efficiency and life of a fuel cell are influenced by pH, so the measurement of pH in a fuel cell has been investigated [1]. There are two common pH sensors on the market, which are the potential pH sensor and conductance pH sensor [2]. The potential pH sensor deduces pH value by referring to the potential difference between electrodes; it is applicable to multiple fluids, but the process is complex and its volume is relatively large [3]. The conductance pH sensor uses an interdigital electrode coated with a dielectric substance to measure conductance; the sensor has a small area and high accuracy and performs better in measuring specific fluids [4]. At present, the pH sensor is large in size and expensive. It is difficult to be embedded in a fuel cell for real-time measurement, and it cannot give consideration to other physical quantities that influence the operation of a fuel cell.

The higher the operating temperature of the fuel cell, the better the performance. This is because increasing the temperature is conducive to improving the electrochemical reaction rate and the proton transfer rate in the electrolyte membrane. Although increasing the operating temperature is conducive to improving fuel cell performance, a proton exchange membrane fuel cell (PEMFC) uses a proton exchange membrane as an organic membrane, and its temperature resistance is limited. Furthermore, considering the membrane water problem, a PEMFC operating temperature cannot be higher than 100 °C; in fact, the general operating temperature is between room temperature and about 80 °C [5]. In addition, the effect of humidity on the fuel cell can increase or decrease the efficiency; excessive humidity can cause the destruction of the proton exchange membrane.

The integrated sensor, using stainless-steel foil as its sensor substrate, can provide better mechanical strength and can measure multiple physical quantities simultaneously; thus, it is paid close attention to for its excellent characteristics [6,7]. The variance in the internal, important physical quantities in the operation of a fuel cell can influence the working efficiency and the life of internal elements directly [8,9,10,11]. Qin found that a water droplet can be driven into the channel sidewall by aerodynamics when the initial water location deviates from the membrane-electrode assembly (MEA) center to form a water corner flow in the flow channel [12].

When the operating temperature of a fuel cell and the relative humidity are too high, the working efficiency of the fuel cell will decrease and shorten the life of internal component [13]. In the past, our research team has successfully developed wired multi-function microsensors for a high-temperature proton exchange membrane fuel cell [14] and a lithium-ion battery [15]. This study uses micro-electro-mechanical systems (MEMS) technology to integrate a micro humidity sensor, micro pH sensor, micro temperature sensor, and micro voltage sensor into a flexible four-in-one microsensor, which has been calibrated. The flexible four-in-one microsensor is embedded in the PEMFC, to measure the local states of pH, temperature, humidity, and voltage in the working fuel cell simultaneously with little effect on efficiency. This study aims at a flexible four-in-one microsensor, which can be embedded in the PEMFC for real-time microscopic diagnosis so as to assist in developing and improving the technology of the fuel cell.

In the past, most of the observed fuel cells used external observation, embedded, or simulation mode. There is single function, large volume, and destructive embedding [16,17,18].

The flexible four-in-one microsensor developed in this study overcomes the limitation of the old external, destructive, or simulation mode. This work uses polymide (PI) film (50 μm) as a flexible substrate. It is small enough to place anywhere between the PEMFC and the flow channel, and no support farm is required. Therefore, the microsensors that are integrated into the fuel cell have the advantage of multi-functionality, high accuracy, high linearity, high sensitivity, extreme flexibility, mass production, and short response time. Thus, the flexible four-in-one microsensor with excellent temperature tolerance and strength is applicable to the electrochemical environment.

## 2. Design of Flexible Four-In-One Microsensor

This study uses micro-electro-mechanical systems technology to integrate a micro pH sensor, micro humidity sensor, micro temperature sensor, and micro voltage sensor into a flexible four-in-one microsensor, which has been calibrated, and the flexible four-in-one microsensor is embedded in the PEMFC to measure the local states of internal pH, temperature, humidity, and voltage in the operation of the fuel cell simultaneously.

### 2.1. Design and Principle of Conductance pH Sensor 

The sensing material is made by mixing conductive polymer and solvent with the adhesion layer made of resin. The conductive polymer is made of polypyrrole (PPY) and polyaniline powder mixed uniformly with butyl glycol ether solvent. The polyaniline is highly sensitive to acid, enhancing the sensitivity of the sensor to pH value. The PPY increases the overall resistance of the sensor and enhances the stability of sensor so that the sensor can work normally in different conditions and similar correction curves can be obtained [13].

The adhesion layer is made of polyvinyl butyral (PVB) resin. The PVB resin is dissolved in butyl glycol ether solvent in proportion and heated to form PVB glue. The uniformly mixed conductive polymer is mixed with the PVB glue and stirred uniformly for 30 min to complete the preparation of the hydrogel.

### 2.2. Design and Principle of Temperature Sensor

**Figure 1:**
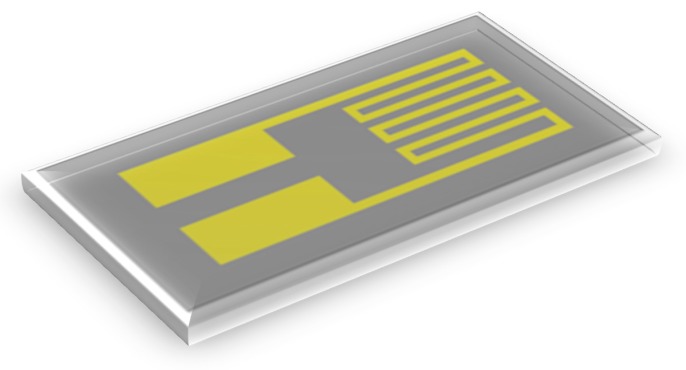
Schematic diagram of temperature sensor.

The micro temperature sensor used in this study is a resistance temperature detector (RTD), the electrode type is serpentine, as shown in Figure 1. The RTD material is Au for its stable chemical properties, simple process, and high linearity [19,20,21].

The sensing principle of the micro temperature sensor is that when the ambient temperature rises, as Au has a positive temperature coefficient (PTC), the resistivity of the RTD increases. This characteristic results from the “temperature coefficient of resistance” (TCR) of a conductor, defined as Equation (1) [15].

(1) $$\alpha =\frac{1}{\rho_{0}}\frac{d\rho }{dT}$$

where *α* is the TCR; *ρ*_0_ is the resistivity at 0 °C.

Therefore, the relation between the resistivity of the conductor and temperature can be expressed as Equation (2).

*R_t_* = *R*_0_ (1 + *α*_1_ Δ*T* + *α*_2_ Δ*T*^2^ + *α*_3_ Δ*T*^3^+⋯) (2)

Δ*T* = *t* − *t*_0_ (3)

where *R_t_* is the resistivity (Ω) at *t* °C; *R*_0_ is the resistivity (Ω) at 0 °C; *α*_1_, *α*_2_, and *α*_3_ are the TCR (%/°C); Δ*T* is the temperature difference (°C) in relation to the reference temperature 0 °C; *t* is the temperature (°C) at *t* °C; and *t*_0_ is the temperature (°C) at 0 °C.

Equation (2) shows that the relationship between the temperature and resistance value of the conductor is nonlinear. But, if the conductor resistance value of the RTD is in the linear range, Equation (2) can be reduced to Equation (4).

*R_t_* = *R*_0_ (1 + *α*_1_ Δ*T*) (4)

### 2.3. Design and Principle of Capacitive Humidity Sensor

**Figure 2:**
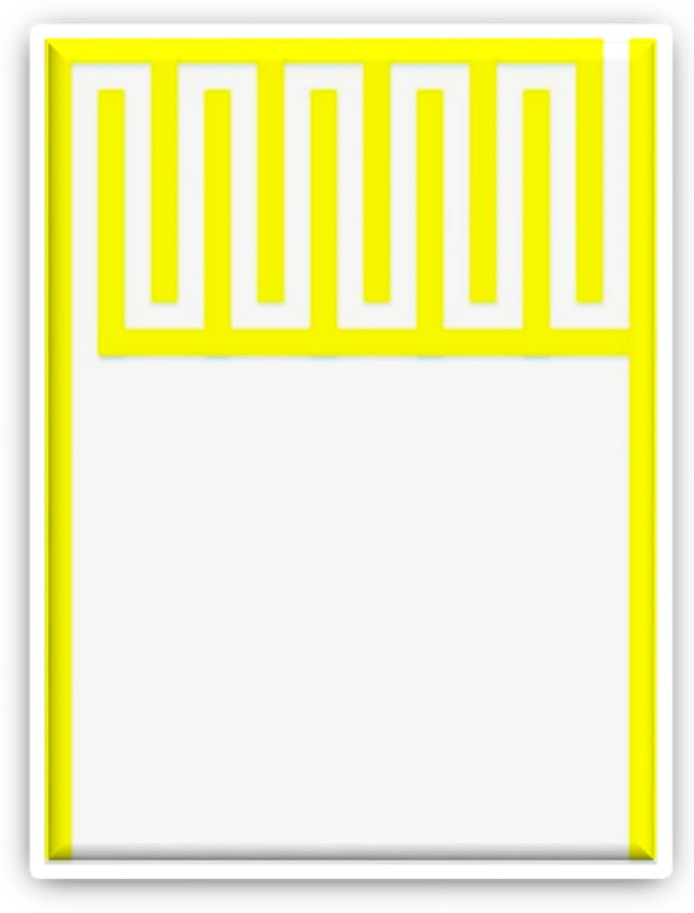
Schematic diagram of capacitive humidity sensor.

As for the capacitive humidity sensor, as shown in Figure 2, the interdigitated electrode is coated with Polyimide (PI) to make sensing film. The humidity can be calculated from the measured capacitance variation of the sensing film in water [22,23,24]. The polymer material of the micro humidity sensor must have the characteristics of a low dielectric constant (about 3–4) and high resistance. Because the dielectric constant of water is 80, the dielectric constant of the material increases as the moisture absorption increases. The value tends to increase as the ambient humidity increases, as shown in Equation (5).

(5) $$C=\varepsilon_{0}\;\varepsilon \left(\%\;RH\right)\frac{A}{d}\;$$

### 2.4. Design and Principle of Voltage Sensor

**Figure 3:**
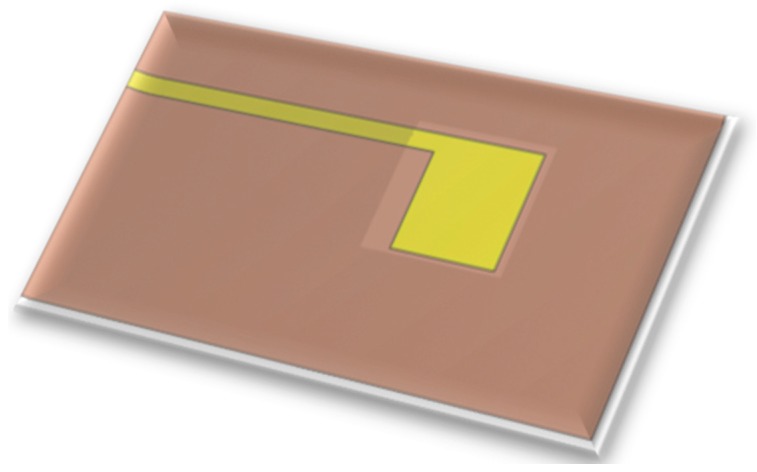
Schematic diagram of voltage sensor.

The voltage sensor is a miniaturized voltage probe, as shown in Figure 3. It is placed on the rib of a fuel cell, and the other portion of the conductor is insulated so that the voltage probe can measure the local voltage accurately [25,26,27].

## 3. Flexible Four-In-One Microsensor Process Development

This study plans to integrate micro pH, temperature, humidity and voltage sensors on a flexible substrate of stainless-steel foil and uses micro-electro-mechanical systems technology to make a flexible four-in-one microsensor with excellent temperature tolerance and strength that is applicable to the electrochemical environment.

The production process of the flexible four-in-one microsensor contains deposition, lithography, photoresist coating, and wet etching. Using micro-electro-mechanical systems technology, (1) the stainless-steel foil substrate is immersed in acetone and methanol and vibrated by ultrasonic cleaner to remove oil, fat, and fine dust from the surface so as to guarantee the adhesion of metal and photoresist in the manufacturing process. The passivation layer is removed by using the solvent of sulfuric acid heated to 80 °C and H_2_O_2_. (2) The PI 7320 is coated as compression resistant insulating layer. (3) In order to enhance the adhesion of Au and PI, the Cr is evaporated as the adhesion layer of Au and PI, and the Au is deposited as a sensing material. (4–5) The sensing pattern of the four-in-one microsensor is defined by photolithography and wet etching process. (6) The PI and hydrogel sensing layer is defined by spin coating photoresist and lithography process. (7) The photoresist is coated as a protection layer to protect the electrode against the impact of fluid in the fuel cell. Finally, the sensing head and signal pins of the sensor are exposed to photolithography for subsequent signal transfer and output. The optical micrograph and real photo of the flexible four-in-one microsensor, the pH, temperature, humidity, and voltage can be measured instantly. There are four functions and many advantages, including high strength, compactness, flexibility, arbitrary placement, and real-time measurement.

The temperature sensing area is 400 μm × 400 μm; the voltage sensing area is 500 μm × 535 μm; and the pH and humidity sensing area are 410 μm × 585 μm.

## 4. Calibration of Microsensor

### 4.1. Calibration of Micro pH Sensor

**Figure 4:**
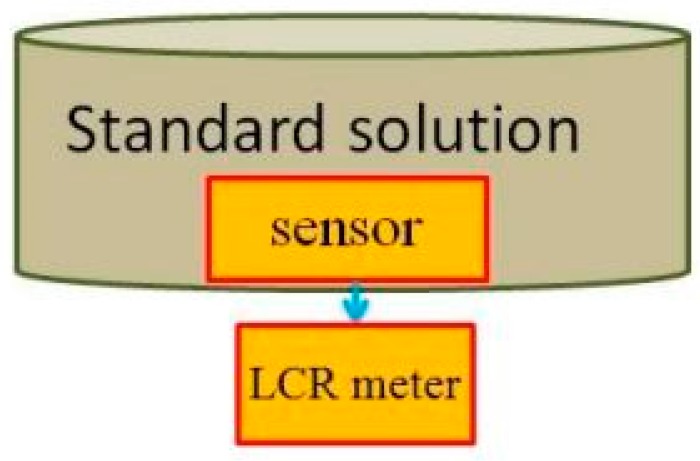
Schematic diagram of calibration of the flexible micro pH sensor.

**Figure 5:**
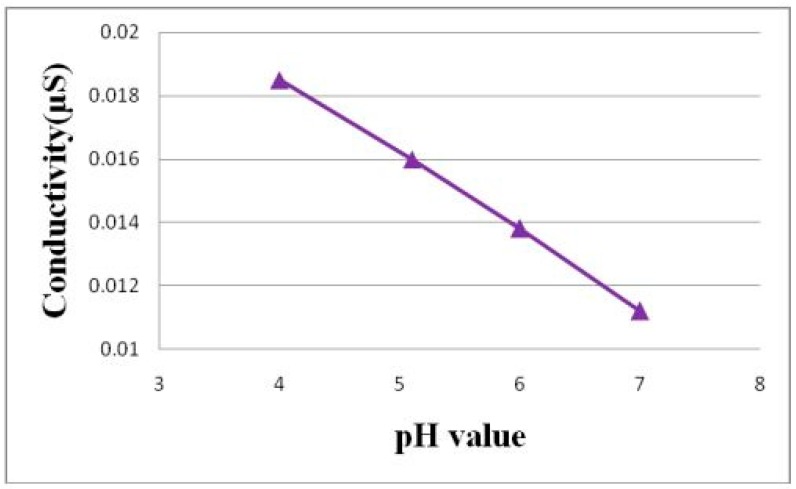
Calibration curve of the flexible micro pH sensor.

Figure 4 is the schematic diagram of the calibration of the flexible micro pH sensor. As the fluid in the low-temperature fuel cell is acidic, the standard solution of pH 7 is the first titer, and the standard solution of pH 6 to pH 4 is the measurement liquid. The calibration method is that the flexible micro pH sensor is immersed in the first titer, and the conductivity is measured by LCR (inductance, capacitance, and resistance) meter [13]. Afterwards, the sensor is immersed in the standard solution of pH 6 to pH 4, and the conductivity is measured so as to obtain the pH value and conductivity correction chart, as shown in Figure 5.

### 4.2. Calibration of Micro Temperature Sensor

**Figure 6:**
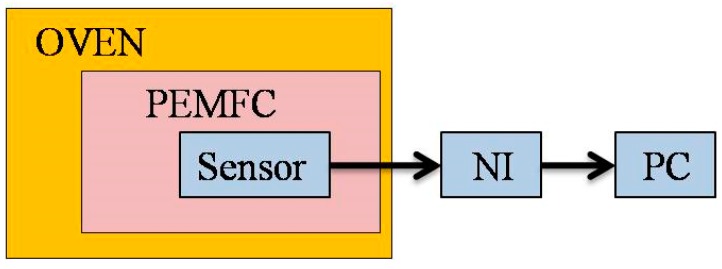
Schematic diagram of calibration of the flexible micro temperature sensor.

**Figure 7:**
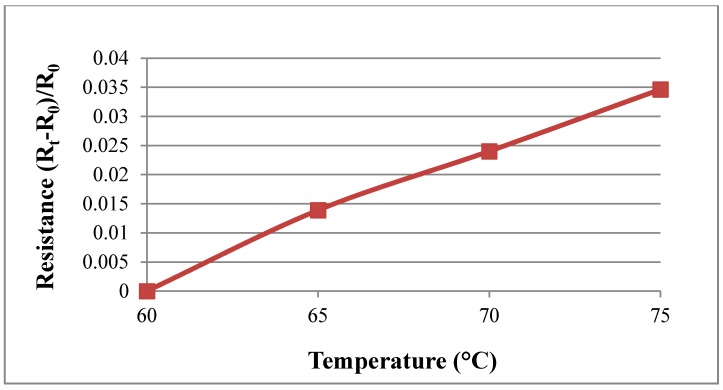
Calibration curves of the micro temperature sensors.

Figure 6 shows the calibration of the micro temperature sensor. The flexible four-in-one microsensor is placed in a temperature-controlled oven and calibrated at a constant temperature. After controlling the temperature to reach a certain temperature and stabilize, the resistance value of the micro temperature sensor under the temperature is obtained by using a data acquisition device (National Instruments, NI, Taipei, Taiwan) and outputting it to the computer to obtain a calibration curve of resistance against temperature. The calibration curve obtained by the micro temperature sensor is in the temperature range of 60–75 °C. The average of three calibrations is shown in Figure 7. Depending on the measurement equipment, the accuracy of the micro temperature sensor is ≤0.5 °C.

### 4.3. Calibration of Micro Humidity Sensor

**Figure 8:**
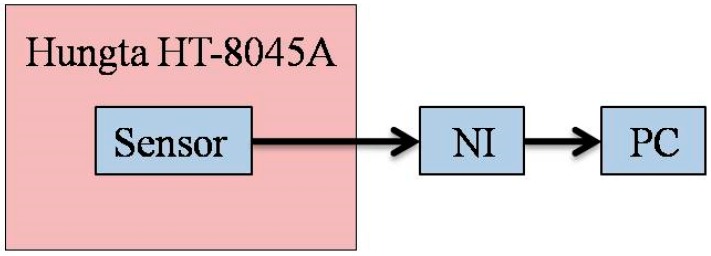
Schematic diagram of calibration of the flexible micro humidity sensor.

**Figure 9:**
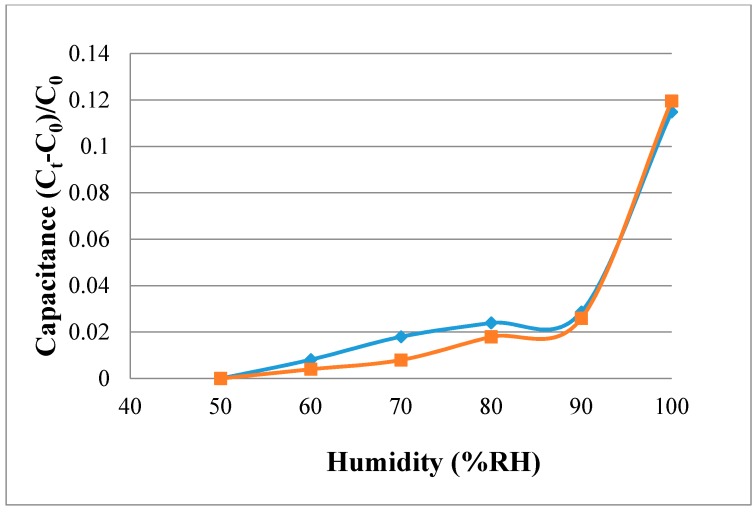
Calibration curves of the micro humidity sensors.

The micro humidity sensor is placed in the Hungta HT-8045A program-controlled constant temperature and humidity chamber for calibration, as shown in Figure 8. The calibration curve of the micro humidity sensor may differ due to the difference in the order of low-humidity and high-humidity calibration, and it may cause hysteresis due to the micro humidity sensor. In the calibration, after the temperature of the constant temperature and humidity chamber is controlled at the operating temperature, the calibration is performed from a relatively low humidity of 50% RH (relative humidity) to a high humidity of 100% RH (absorption) and from a high humidity of 100% RH to a low humidity of 50% RH (desorption), and at the same time, the data acquisition device NI captures the capacitance value of the micro humidity sensor under different conditions and sends it to the computer. Figure 9 shows the calibration curve obtained after the micro-moisture sensor has been calibrated three times and averaged. The calibration curves of the micro humidity sensor are not linear. Ideally, the sensor should exhibit no hysteresis and nonlinearity, which phenomena are common in humidity sensor applications based on adsorption. Depending on the measurement equipment, the accuracy of micro humidity sensor is ≤3% RH.

## 5. Local Real-Time Microscopic Monitoring in Fuel Cell

**Figure 10:**
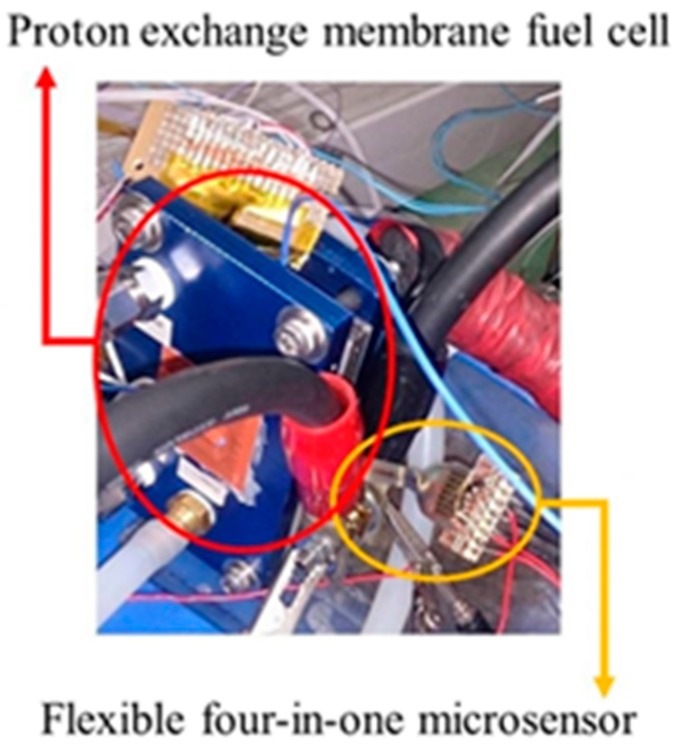
Stereogram of fuel cell embedded with flexible four-in-one microsensor.

**Figure 11:**
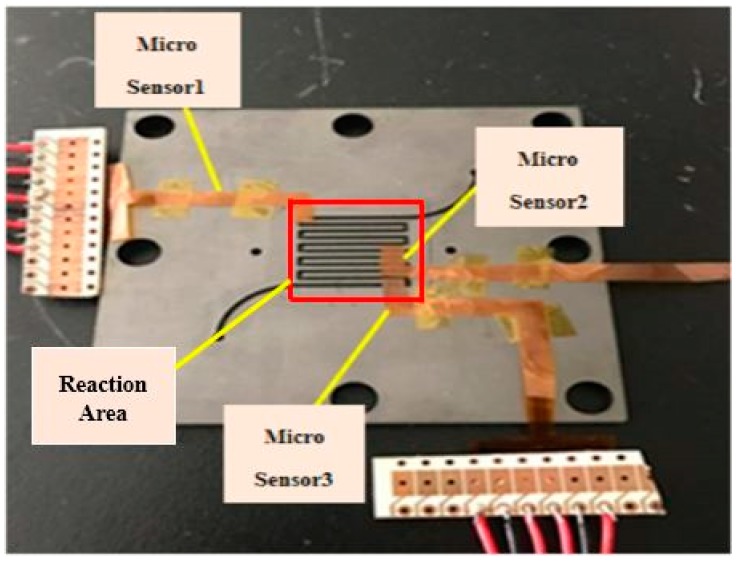
Schematic diagram of flexible four-in-one microsensor embedded in a cathode channel plate.

After the reliability test, the flexible four-in-one microsensor can be embedded in the fuel cell for real-time diagnosis, as shown in Figure 10. This study uses a 10 cm^2^ (reaction area: 6.25 cm^2^) fuel cell for testing. In order to stabilize the fuel cell performance and to avoid the fuel gas leaking during the fuel cell testing process—the graphite bipolar plate breaks if the closing pressure is too high, and the gas leaks if the closing pressure is too low—this study uses closing pressure of 25 kg/cm^2^ to close the cell uniformly, as shown in Figure 11. The fuel cell operating environment is set to 60 °C. The 75 °C humidified gas of anode flow rate (H_2_, 210 cc/min) and cathode flow rate (Air, 1300 cc/min) are given. In the constant current test (8 A, 16 A) conditions, the data of local temperature, voltage, and humidity changes in the fuel cell are extracted by using a NI PXI 2575 data acquisition unit, and the pH variation is measured.

The embedded flexible four-in-one microsensors inside the fuel cell can cover the reaction area of PEMFC to prevent the gas from reacting with the covered PEMFC. However, as the size of the four-in-one microsensor is too small (0.03 cm^2^), the area of the four-in-one microsensor accounted for only 1.44% of the PEMFC reaction area. The main cause of the loss in performance is the location of the four-in-one microsensor, because it could resist the pass of the protons and reduce the reaction area of the fuel cell.

**Figure 12:**
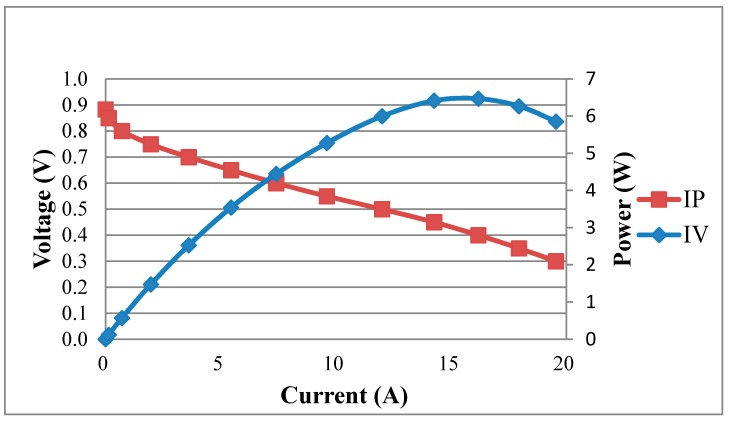
Performance curve of a fuel cell embedded with the flexible four-in-one microsensor.

**Figure 13:**
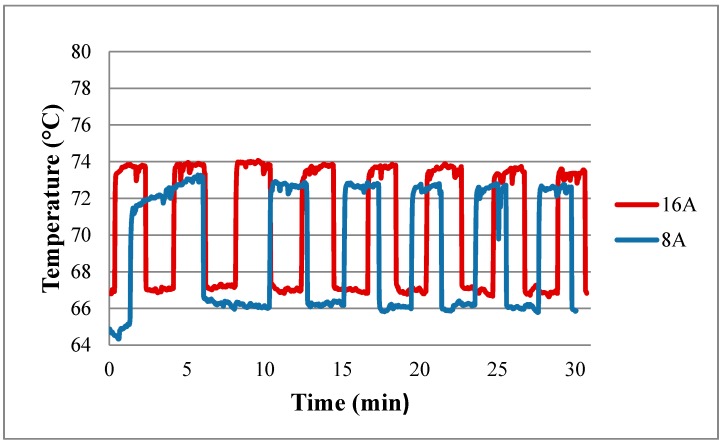
Real-time measurement of temperature.

Figure 12 shows the performance curve of a fuel cell embedded with the flexible four-in-one microsensor at working temperature of 60 °C. The real-time measurement of the local temperature in the fuel cell during the constant current (8 A, 16 A) output is shown in Figure 13. The cell operating temperature is set as 60 °C, but the internal electrochemical reaction is drastic, and the internal temperature of cell changes. The maximum temperature exceeds 70 °C. Also, in the high current (16 A) operating condition, the measured internal temperature is higher than that in low current (8 A) operating condition.

**Figure 14:**
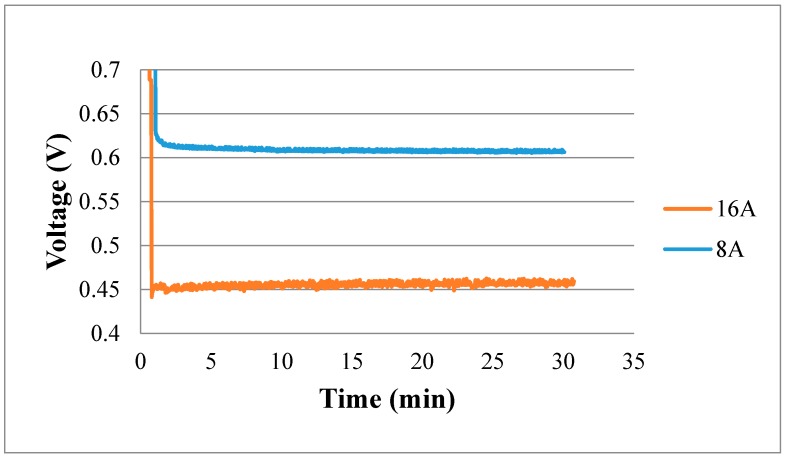
Real-time measurement of voltage.

Figure 14 shows the real-time measurement of the internal voltage. In the higher constant current loading condition (16 A), the voltage curve has greater oscillation. This means that the internal electrochemical reaction is drastic in the high current loading condition so that the voltage changes drastically.

**Figure 15:**
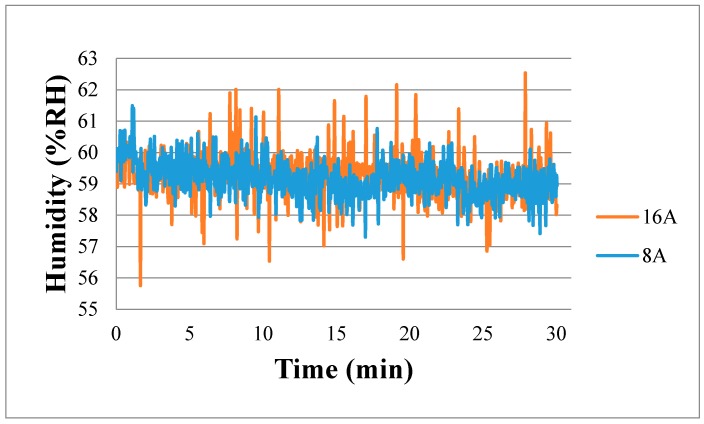
Real-time measurement of relative humidity.

Figure 15 shows real-time measurement of local relative humidity in the fuel cell. It is observed that in the high current (16 A) operating condition, the relative humidity is unstable and fluctuating largely, as the electrochemical reaction is drastic. In the high current (16 A) operation, the relative humidity is a little higher than the relative humidity in low current (8 A) operation. This is because when the chemical reaction in the fuel cell is drastic, the relative humidity is higher.

**Figure 16:**
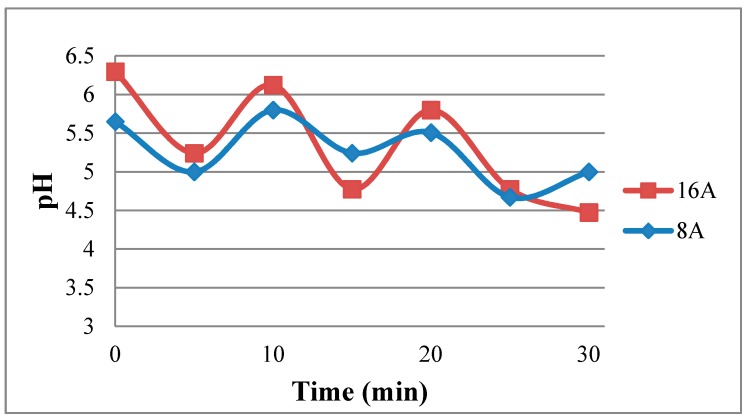
Real-time measurement of pH.

Figure 16 shows the real-time measurement of local pH in the fuel cell. It is observed that the pH in the high current (16 A) operating condition is higher than that in low current (8 A) operation. It may be because the internal chemical reaction is drastic and rapid in the high current operation, leading to higher humidity, and higher humidity increases local pH. As the fuel cell operating time extends, the internal pH of cell decreases. This phenomenon may result from the accumulation of acidic fluid in the fuel cell.

## 6. Conclusions

The flexible four-in-one microsensor can be embedded in the fuel cell to measure the local temperature, humidity, and voltage data in specific positions instantly. In the past, only the pH at the fuel cell outlet/inlet could be measured, or only one physical quantity could be measured. The flexible four-in-one microsensor developed in this study overcomes the limitations of the old external, destructive, and simulation modes. This work uses polymide (PI) film (50 μm) as a flexible substrate. It is small enough to place anywhere between the PEMFC and the flow channel, and no support farm is required. Therefore, the microsensors that are integrated into the fuel cell have the advantage of multi-functionality, high accuracy, high linearity, high sensitivity, extreme flexibility, mass production, and short response time. Thus, the flexible four-in-one microsensor with excellent temperature tolerance and strength can be applicable to the electrochemical environment.
